# Analysis of the Metabolic and Structural Changes in *Ulmus pumila* ‘Zhonghua Jinye’ Leaf Under Shade Stress

**DOI:** 10.3390/plants14182868

**Published:** 2025-09-15

**Authors:** Yichao Liu, Yongtan Li, Ning Wang, Lihui Zuo, Yang Zhou, Ping Li, Shijie Wang, Shuxiang Feng, Shufang Yan, Yinran Huang, Minsheng Yang

**Affiliations:** 1 Hebei Forestry and Grassland Science Research Institute, Shijiazhuang 050000, China; 2 College of Forestry College (Landscape and Tourism), Hebei Agricultural University, Baoding 071000, China; 3 College of Landscape and Ecological Engineering, Hebei University of Engineering, Handan 056000, China

**Keywords:** adaptation, regreening, self-regulation, shade stress, *Ulmus pumila* ‘Zhonghua Jinye’

## Abstract

*Ulmus pumila* L. ‘Zhonghua Jinye’ is a plant variety with colourful leaves that is widely used in landscaping. In our study, the leaves of *U. pumila* ‘Zhonghua Jinye’ fade and turn green under light (LT, 45%), moderate (MD, 70%) and high (HG, 95%) shading treatment, reducing its ornamental value. However, the mechanism underlying this adaptation to shade is poorly understood. The objective of this study was to elucidate the mechanism of physiological, cellular microstructural and transcriptional changes involved in leaf regreening in *U. pumila* ‘Zhonghua Jinye’. Our results showed that the pigment content of *U. pumila* ‘Zhonghua Jinye’ leaves increased under shade stress, with a corresponding colour change from yellow to dark green. Thus, *U. pumila* ‘Zhonghua Jinye’ adapted to shade stress by increasing leaf pigment and chlorophyll content. Transmission electron microscopy showed that thylakoid stacking in the grana lamellae changed significantly from a loose state to a closely packed structure under shaded conditions. Because plant pigments were located mainly in thylakoids, this closer stacking increased photosynthetic efficiency and pigment accumulation. RNA sequencing analysis showed that *Lhcb1*, a key thylakoid membrane gene, was upregulated under shade, which promoted thylakoid stacking and light absorption. In the chlorophyll synthesis pathway, haeme metabolism was inhibited, increasing protoporphyrin IX flow to the chlorophyll pathway and promoting the synthesis of chlorophyll a/b. The simultaneous upregulation of plant hormone-related genes promoted an increase in plant leaf area, improving the light energy utilisation ratio. This study is the first to report the self-regulatory mechanism that leads to colour change in *U. pumila* ‘Zhonghua Jinye’ under shade stress and provides a theoretical basis for the cultivation of tree species with colourful leaves.

## 1. Introduction

Elms (*Ulmus*) grow in temperate, warm temperate, and subtropical climates and have long been used as a source of high-quality hardwood and have high resistance to drought, cold and salt stress. *Ulmus pumila* ‘Zhonghua Jinye’ is a natural mutant of *Ulmus pumila* L. that is distributed throughout most of China. This variety has bright golden-yellow leaves and is therefore of great ornamental value in landscape gardening [1,2].

Photosynthesis is the process through which green plants use carbon dioxide, water, and light energy to produce organic substances and release oxygen. Light is indispensable for photosynthesis in plants, which provides energy for growth and development, and carbon was as main structural material. Plant adaptability to changes in the light environment largely determines their distribution patterns and species richness. Plants with colourful leaves and flowers are valued for their visual enrichment of urban landscapes. However, most colourful plants are extremely sensitive to light intensity. In trees with colourful leaves, changing leaf colour is a widespread phenomenon that reduces their ornamental value, presenting a major problem in landscaping [3,4,5]. In many such plant species, leaves exhibit colour fading or greening under light stress conditions; these include *Acer negundo* ‘Aurea’, *U. pumila* ‘Zhonghua Jinye’, and *Berberis thunbergii* var. *Atropurpurea* [6,7,8,9]. In *U. pumila* ‘Zhonghua Jinye’, leaves turn pale yellow under moderate shade stress, and dark green under severe shade stress [7]. However, little is known about the molecular mechanism of these leaf colour changes under shade stress. In this study, we explored the mechanism of colour changes in yellow leaves of *U. pumila* ‘Zhonghua Jinye’ by examining transcriptomic, physiological and phenotypic variation under different shading conditions. Our results provide information for the future maintenance and development of colourful trees.

## 2. Materials and Methods

### 2.1. Materials and Treatments

In this study, we collected branch samples from *U. pumila* ‘Zhonghua Jinye’ trees that were managed normally. Normal growth clones were selected and planted in pots for follow-up experiments. The gene transcriptional, physiological and phenotypic differences in *U. pumila* ‘Zhonghua Jinye’ leaves were studied under CK (0%, 70,000 lux), light (LT, 45%, 38,500 lux), moderate (MD, 70%, 17,500 lux) and high (HG, 95%, 3500 lux) shade stress. After shading for 15 days, fresh leaves were collected when the treated plants showed obvious and consistent effects of shading. Plant materials were separated into three groups for chloroplast observation by electron microscopy, physiological tests and preservation in a refrigerator at −80 °C for subsequent transcriptional experiments.

### 2.2. Pigment and Leaf Colour Determination and Ultra-Structural Analyses

To compare pigment content among treatments, we performed organic solvent extraction as described previously [10]. Leaf samples were cut into 1 mm × 1 mm pieces, fixed with 2.5% glutaraldehyde solution, and stained with 3% uranyl acetate (*w*/*v*) and lead citrate. The sections were examined and photographed using a JEM-2000EX transmission electron microscope (JEOL, Tokyo, Japan) with an acceleration voltage of 80 kV. To accurately determine colour changes, we used a colourimeter (CR-400, Minolta, Tokyo, Japan) to obtain digital leaf colour values.

### 2.3. RNA Extraction, Library Construction and Sequencing

All materials including CK, LT, MD and HG. were randomly selected from each treatment for RNA extraction. The total RNA of leaves treated under different shade stress for 15 days was extracted using the EASYExPLUS Plant RNA Kit (Cyrus, Beijing, China), a Qubit 2 fluorometer (Thermo Fisher Scientific, Waltham, MA, USA), and an Agilent 2100 bioanalyser (Agilent Technologies, Santa Clara, CA, USA) to detect the purity, concentration and integrity of the RNA samples. A library was constructed from qualified samples and quantified accurately using quantitative polymerase chain reaction (qPCR) to ensure quality. After quality testing, an Illumina HiSeq 2500 device (Illumina, San Diego, CA, USA) was used for high-throughput sequencing.

### 2.4. Illumina Sequencing, Assembly, and Functional Annotation

Adapter and primer sequences and low-quality data were removed from the raw data to obtain high-quality reads. The Benjamini–Hochberg approach was used to screen differentially expressed genes (DEGs) to correct *p* values obtained from previous hypothesis tests. DEGs were screened according to the criteria of false discovery rate (FDR) < 0.01 and fold change (FC) > 2 [11]. The sequencing data were assembled using the Trinity v2.15.1 software and unigene sequences were compared using the non-redundant (Nr), Swiss-Prot, Clusters of Orthologous Groups of proteins (COG), EuKaryotic Orthologous Groups (KOG), Gene Ontology (GO), and Kyoto Encyclopedia of Genes and Genomes (KEGG) databases to obtain annotation information [12,13,14,15,16,17,18,19].

### 2.5. The Trend, GO and KEGG Pathway Enrichment of DEG

Gene expression pattern analysis of multiple samples was conducted to cluster genes with similar expression patterns. For DEG expression pattern analysis, the expression data for each sample were normalised as log_2_(LT/CK), log_2_(MD/CK) and log_2_(HG/CK), and then clustered using the STEM v1.3.13 software [20] using the following parameters: maximum unit change in model profiles between time points = 1, maximum number of output profiles = 20 (similar profiles were merged), and minimum FC ratio of DEGs ≥ 2.0. Cluster profiles with *p* ≤ 0.05 were considered to be significant. The DEGs in each profile were subjected to GO and KEGG pathway enrichment analysis. GO terms or pathways with Q values ≤ 0.05 following *p* calculation through hypothesis testing and FDR correction were defined as significantly enriched.

### 2.6. Real-Time qPCR Verification

Total RNA was extracted using the EASYExPLUS Plant RNA Kit (Cyrus). Reverse transcription of the first cDNA chain was performed using a reverse transcription kit (Kangwei, Shenzhen, China). cDNAs synthesised by reverse transcription were used as the template, and 2× SYBR Green qPCR Mix (Thermo Fisher Scientific) was used for fluorescence qPCR, with three biological replicates and three technical replicates. The reaction system (20 μL) comprised 10 μL 2× SYBR qPCR Mix, 0.5 μL forward primer (10 μM), 0.5 μL reverse primer (10 μM), 1 μL template, and 8 μL ddH_2_O. The reaction procedure was as follows: pre-degeneration at 95 °C for 5 min, degeneration at 95 °C for 10 s, and renaturation at 55 °C for 30 s. Fluorescence qPCR primers were designed according to the sequence information of the target genes using the Primer Premier 5 (Premier Biosoft, San Francisco, CA, USA) (Table S1).

## 3. Results

### 3.1. Effects of Shade Stress on Phenotype and Physiological Indices

**Figure 1:**
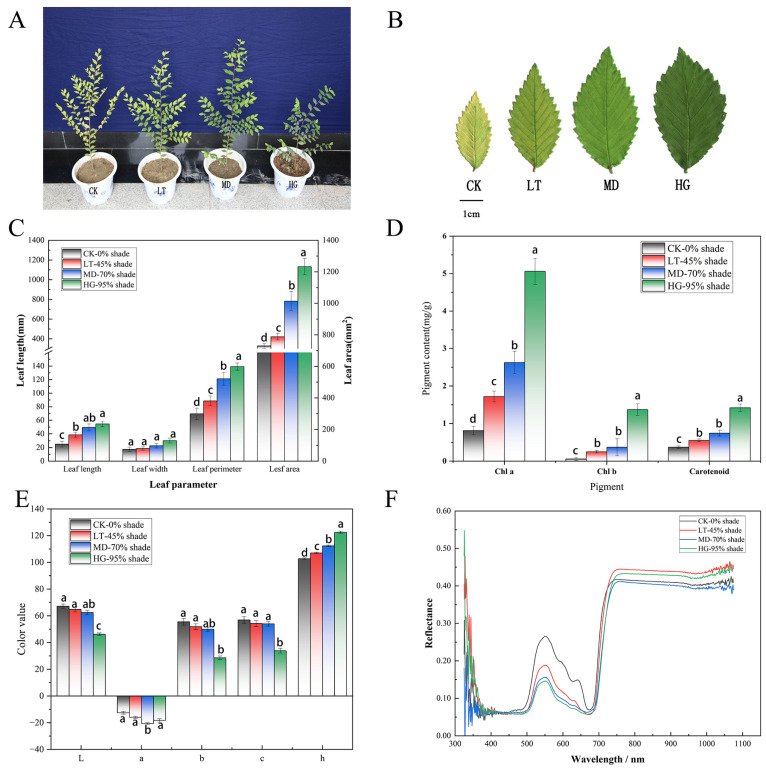
Phenotypic changes after shading treatment of *U. pumila* ‘ZhonghuaJinye’. (**A**) Changes of plant growth after shading stress treatment. (**B**) Phenotypic changes of leaves after shading stress treatment. (**C**) Changes of leaf size related parameters under Shading Stress. (**D**) Changes of leaf pigment content under Shading Stress. (**E**) Changes of leaf colour parameters under Shading Stress. (**F**) Changes of leaf reflectance spectra under Shading Stress. Note: CK, LT, MD, and HG indicate 0%, 45%, 70%, and 95% shade, respectively (**A**,**B**). The left side of the vertical axis represents the length of the leaves, and the right side represents the area of the leaves (**C**). The different letters in the bar chart indicate the significance of the comparison difference between groups (*p* < 0.05) (**C**,**D**).

After shading for 15 days, the phenotypic traits of *U. pumila* ‘Zhonghua Jinye’ changed significantly. The LT and MD treatments showed no significant changes in plant growth or height (Figure 1A), whereas in the HG treatment, plant height was significantly inhibited and horizontal branch growth was enhanced, resulting in wide-angled branches. The most obvious change under shade stress was in leaf colour and size. Under increasing shade stress, leaves turned from yellow to green, gradually deepening in colour, and leaf area increased (Figure 1B). Leaf length increased in all treatments, by 23.46% (LT), 62.96% (MD) and 82.72% (HG) (Figure 1C), respectively. Leaf length, area, and perimeter also showed increasing trends; the greatest increase was observed in leaf area, by 0.35% (LT), 154.38% (MD), and 270.18% (HG) (Figure 1C). Leaf pigment analyses showed that chlorophyll a (Chla), chlorophyll b (Chlb), and carotenoid content in leaves increased significantly with increasing shade stress (Figure 1D). Chlb showed the greatest relative change, increasing 27-fold, followed by Chla (5-fold), and carotenoid. The proportions of pigments also changed significantly; compared with the control (CK), (Chla + Chlb)/carotenoid and Chla/carotenoid ratios increased as shade stress increased, whereas the Chla/Chlb ratio decreased sharply from 13.85 (CK) to 3.70 (HG) (Figure 1E). With increasing shade stress, reflectivity decreased within the visible wavelength range (400–800 nm), which was consistent with the pigments content analysis results (Figure 1F). Thus, higher pigment content was associated with a stronger ability to absorb light energy, which led to a decrease in reflectivity. Changes in the content and proportion of pigments ultimately led to leaf colour changes. Because visually observed colour changes depend on the observer’s colour sensitivity, we used a colourimeter (CR-400, Minolta) to digitise five parameters of leaf colour: luminosity (L*), red/green balance (a*), yellow/blue balance (b*), chroma (C*), and hue (*h*). L*, a*, C*, and b* values decreased with increasing shade stress, whereas *h* increased; this suggested that leaf colour decreased under enhanced shading and green colour gradually strengthened. These results indicate that *U. pumila* ‘Zhonghua Jinye’ responds to shade stress by increasing leaf area and branching angle to increase light area, while increasing pigment content and relative Chla + Chlb content to maintain photosynthetic efficiency.

### 3.2. Chloroplast Thylakoid Structural Changes

**Figure 2:**
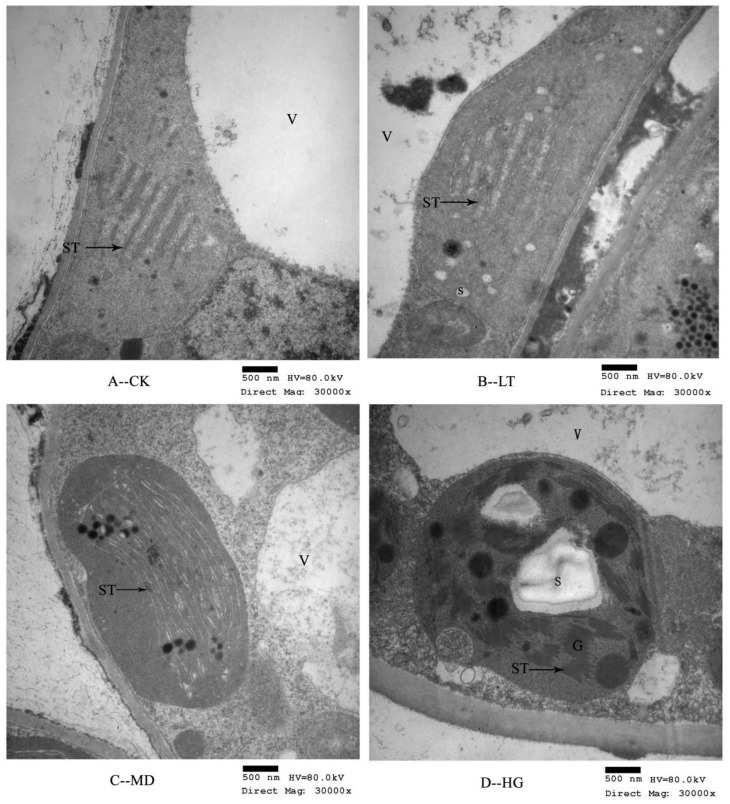
Chloroplast ultrastructure under different shading treatments. Note: (**A**–**D**) show the microstructure of chloroplasts under different shading stress (30,000×). G—Granum; ST—Stoma thylakoid; S—Starch; V—Vacuole.

To further explore the colour changes of *U. pumila* ‘Zhonghua Jinye’ under shade stress, we observed chloroplast microstructures using transmission electron microscopy. Studies of *U. pumila* ‘Zhonghua Jinye’ have reported incomplete chloroplast development and a lack of chloroplasts in many mesophyll cells under normal circumstances. We observed that chloroplast membranes were thin and had damaged edges, indicating imperfect development of the chloroplast membrane system [8]. Under normal conditions, fewer thylakoid grana slice layers were observed (stack failure; Figure 2A). Total thylakoid numbers were lower, with most found in the chloroplast stroma. However, under shade stress, the number of grana thylakoids increased significantly (Figure 2B–D) and the number of stacked layers increased, with slight crosslinking structures among grana. This phenomenon was more obvious under severe shade conditions; the grana lamellae were stacked tightly, forming a dense network structure among grana through ST crosslinking, resulting in thylakoid stacking similar to that observed in normal *U. pumila*. Thylakoids are pigment carriers, and their stacking structure directly affects pigment accumulation and photosynthetic efficiency.

### 3.3. Transcriptome Analysis

After sequencing quality control and the removal of sequencing primers, joints, and other low-quality reads, 32.17 Gb of clean data were obtained. Guanine–cytosine (GC) content ranged from 45.19% to 46.28%, and the Q30 bases of all samples exceeded 92.08%, which met the requirement for the follow-up test. The Trinity software was used to assemble the sequenced data. A total of 2,616,032 contigs (average length, 63.38 bp; N50 length, 49 bp) were assembled and 118,823 transcripts and 62,945 unigenes were obtained. The N50 lengths of transcripts and unigenes were 1998 and 1610 bp, respectively. The percentages of transcripts and unigenes over 1 kb were 46.60% and 28.0%, respectively, indicating that the experimental data assembly had good integrity.

Unigene sequence alignments were matched with the Nr, Swiss-Prot, GO, COG, KOG, and KEGG databases using the BLAST v2.14 software, and KEGG orthology results from unigenes matched in KEGG were obtained using the KOBAS 2.0 web server. Amino sequences were predicted, and unigenes were matched in the Pfam database using the HMMER v3.3.2 software to obtain annotations. BLAST and HMMER produced E values of 1 × 10^−5^ and 1 × 10^−10^, respectively, and 33,023 annotated unigenes (52.46% of the total) were obtained. The highest and lowest numbers of annotated unigenes were obtained from the Nr (48.93%) and COG (19.05%) databases, respectively (Table 1).

**Figure 3:**
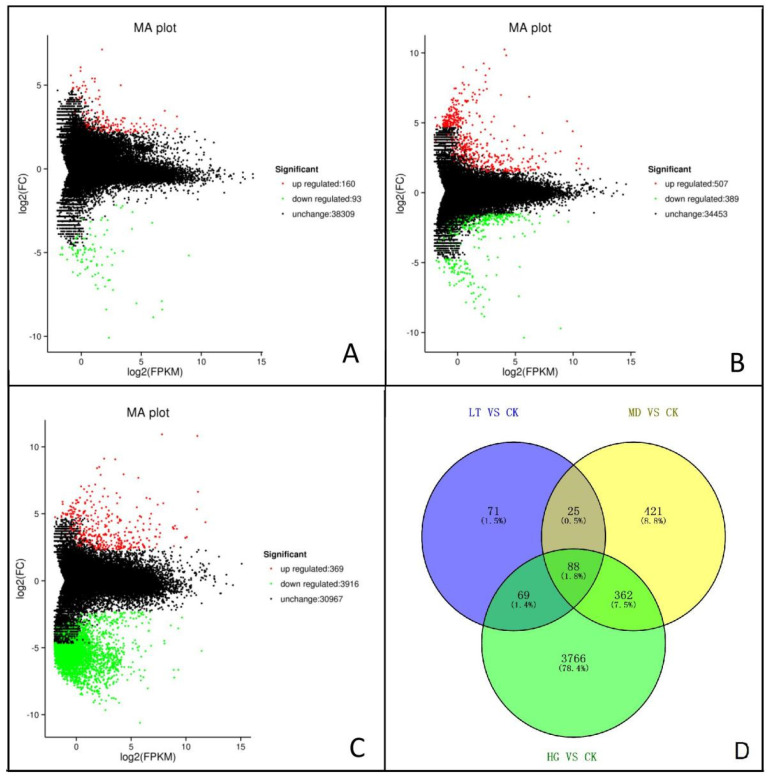
MA map and Venn diagram of differentially expressed genes. Note: (**A**–**C**) denote differentially expressed genes in LT vs. CK, MD vs. CK, and HG vs. CK, respectively. (**D**) Venn diagram of differentially expressed genes. In the graph, each point represents a gene, abscissa is a value: log_2_(FPKM), the ordinate is M value: log_2_(FC). The green and red dots represent the significantly differentially expressed genes, where green represents gene expression downregulation and red represents upregulation; black dots represent genes with no significant difference.

An MA plot of DEGs showed that their number increased gradually with increased shade stress. Compared with the CK, there were 253 DEGs in the LT treatment, among which 163 were upregulated and 90 were downregulated (Figure 3A), whereas the MD treatment produced 896 DEGs, among which 507 were upregulated and 389 were downregulated (Figure 3B) and the HG treatment produced 4285 DEGs, including 369 upregulated and 3916 downregulated genes (Figure 3C). Venn diagram analysis showed a large number of unique DEGs in different treatments, whereas only 88 DEGs were common among treatments (Figure 3D).

### 3.4. GO Functional Classification of DEGs

**Figure 4:**
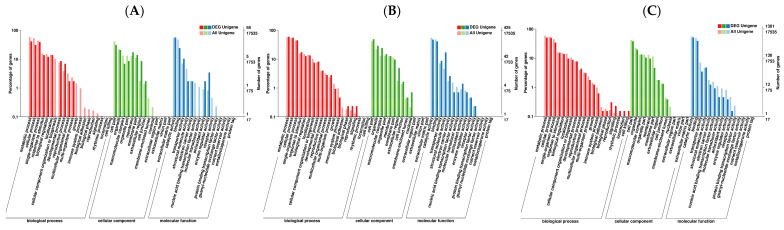
The GO function enrichment. Note: (**A**–**C**) denote The GO function enrichment of differentially expressed genes in LT vs. CK, MD vs. CK, and HG vs. CK, respectively.

The transcriptome of *U. pumila* ‘Zhonghua Jinye’ under shade stress was constructed based on GO function enrichment analysis results. In total, 58, 425 and 1301 differentially expressed unigenes were annotated in samples from the LT, MD and HG treatments, respectively (Figure 4). Among biological processes, DEGs from the three shade stress treatments correlated strongly with metabolic processes, single-organism processes, cellular processes, stimulus responses, biological regulation, and localisation. Among cellular components, the DEGs were associated mainly with cell parts, organelles, and the macromolecular complex under shade stress. All shade treatments affected cells and organelles, whereas the LT treatment had a greater effect on cell membrane structure and the MD and HG treatments had greater effects on polymer complex synthesis. Among molecular functions, DEGs in samples from the LT treatment were related to catalytic activity, binding, and transporter activity, whereas those from the MD and HG treatments were related to catalytic activity, binding, and structural molecule activity. Thus, shade stress may have greater impacts on enzyme activity, transporter activity, electron transport, and transcription in *U. pumila* ‘Zhonghua Jinye’.

### 3.5. Trend Analysis of DEGs

**Figure 5:**
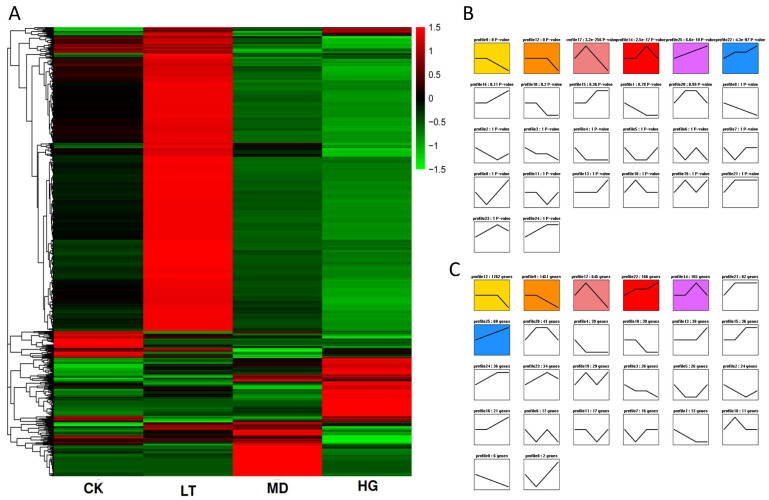
Trend analysis of all profiled genes by gene member and *p* value. Note: (**A**) Heat map of differentially expressed genes. (**B**) gene expression is indicated according to the number of genes. (**C**) gene expression is indicated according to the *p* value. Coloured trend blocks show significant enrichment (*p* < 0.05); similar trends have the same colour. Trend blocks without colour are not significantly enriched.

Our analysis of all DEGs showed significant differences in gene expression patterns among the shade stress treatments (Figure 5A). To examine the mechanism of the adaptation to shade of *U. pumila* ‘Zhonghua Jinye’, we performed trend analysis of 4802 screened DEGs, which we divided into 26 profiles (Figure 5B). The numbers of DEGs per profile ranged from 3 to 1763; profiles 8 and 12 contained the lowest and highest numbers of DEGs, respectively. Six profiles (9, 12, 14, 17, 22 and 25) were significantly enriched (*p* < 0.05); these profiles were the focus of subsequent analysis of re-programming results (Figure 5C). Our previous analysis showed an increase in pigment content with increasing shade stress. Gene expression trends in profiles 22 and 25 were consistent with these changes in pigment content; therefore, we focused our analysis on these profiles. GO database analysis showed similarities among GO subcategory results of the two significantly enriched profiles (Figure S1). Among biological processes, the profiles showed more changes in cellular, metabolic, and single-organism processes. Among cellular components, the profiles showed more changes in cells, cell parts, and organelles. Among molecular functions, the most active proteins were related to binding and catalytic activity. These results indicate that plants influenced protein expression and function through signal transduction; thus, a series of effects occurs in the nuclear structure and organelles during leaf regreening in *U. pumila* ‘Zhonghua Jinye’.

Genes from profiles 22 and 25 were matched in the GO database, and the gene set was found to be significantly enriched on GO:0009505 (cell wall), GO:0008810 (cellulase activity), GO:0006949 (syncytium formation), GO:0016021 (integral component of the membrane), GO:0031224 (intrinsic component of the membrane), GO:0009522 (photosystem I), GO:0009523 (photosystem II), GO:0016157 (sucrose synthase activity), and GO:0005985 (sucrose metabolic process). These GO terms mainly involve sucrose metabolism, as well as cellular membrane and photosystem structure, indicating that cellular structure varies greatly during leaf colour changes in *U. pumila* ‘Zhonghua Jinye’.

### 3.6. KEGG Metabolism Pathway Analysis

DEGs produced by the different shade treatments were classified according to KEGG annotation results. The treatments produced different re-programming mechanisms, and the number of KEGG annotations increased with increasing shade stress (Figure S2). Under LT stress, the pathway related to metabolism accounted for a large proportion of DEGs (*n* = 23, 60.05%), such as Arginine and proline metabolism, Cysteine and methionine metabolism, Glycine, serine and threonine metabolism, Histidine metabolism, Lysine degradation Phenylalanine metabolism. Under MD stress, the pathways enriched with DEGs were related to genetic information processing (*n* = 125, 35.11%), such as Ribosome, Protein processing in endoplasmic reticulum. And the DEGs were related to metabolism (*n* = 111, 31.18%) such as Carbon metabolism, Oxidative phosphorylation, Pentose and glucuronate interconversions. Under HG stress, the pathways involved were related mainly to genetic information processes (*n* = 519, 30.57%) such as Ribosome, Protein processing in endoplasmic reticulum. Then the pathways enriched with DEGs were related to metabolism (*n* = 494, 29.09%) such as Carbon metabolism, Purine metabolism. Moreover, large proportions of DEGs were also related to cellular processes (*n* = 239, 14.08%) such as Oocyte meiosis, Cell cycle. And the DEGs were related to organismal systems (*n* = 233, 13.72%) such as Insulin signalling pathway, Oxytocin signalling pathway. Then, the DEGs were also related to environmental information processing (*n* = 212, 12.49%) such as AMPK signalling pathway, PI3K-Akt signalling pathway.

A comparison of the 15 most common pathways across all shade treatments showed that the number of DEGs increased mainly along the same pathway as shade stress increased (Table 2). The carbon metabolism pathway had the highest number of DEGs across all treatments (*n* = 2, 18 and 66, respectively), indicating that the expression of carbon metabolism-related genes was sensitive to illumination. The cutin, suberin, and wax biosynthesis pathways had the lowest numbers of DEGs across all shade treatments (*n* = 3, 2, and 5, respectively), indicating that shade stress had little influence on biosynthesis.

**Figure 6:**
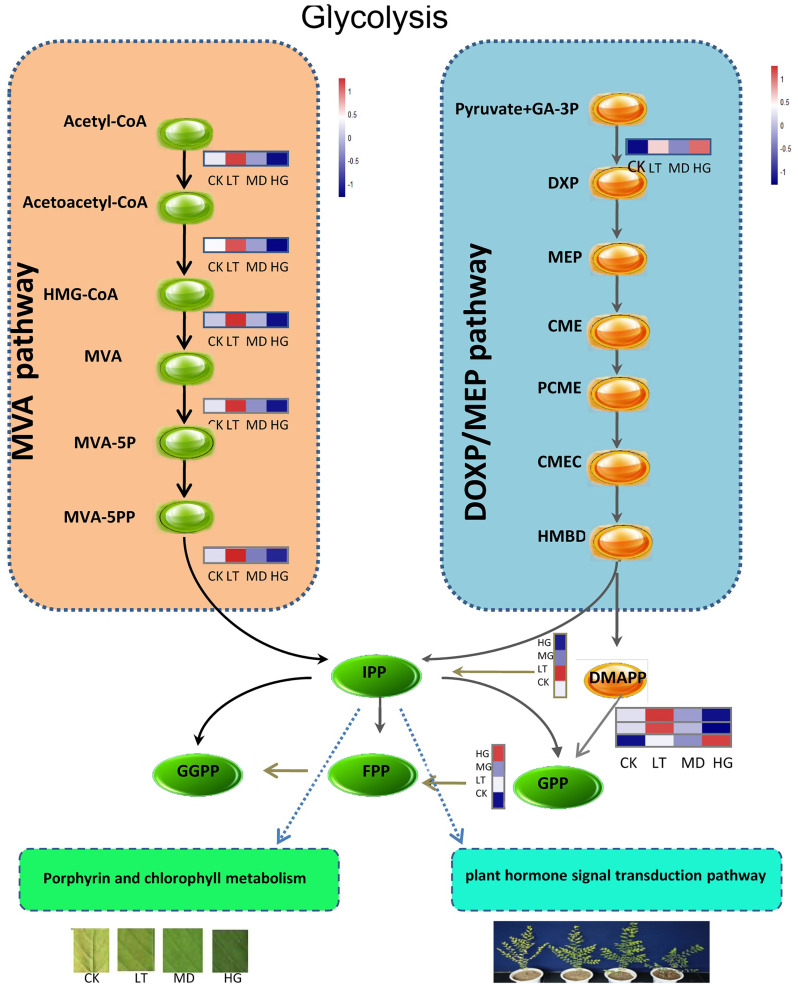
The pathway of flavonoids biosynthesis.

Terpenoids are important secondary metabolites in plants, which play important roles in plant growth and development and abiotic stress resistance. Plants have two terpenoid biosynthetic pathways, the mevalonate (MVA) and 2C-methyl-D-erythritol-4-phosphate (DOXP/MEP) pathways. In this study, these pathways showed increasing trends under shade stress, with LT showing an increase in the MVA pathway and MD and HG showing an increase in the DOXP/MEP pathway. Genes related to the key rate-limiting enzyme hydroxymethylglutaryl-CoA reductase (NADPH) [EC:1.1.1.34] (HMGR) and mevalonate kinase (MVK) in MVA were upregulated under shade stress, as was the primary enzyme 1-deoxy-D-xylulose-5-phosphate synthase [EC:2.2.1.7] (DXS) (Figure 6). Their joint action increased the content of isopentenyl diphosphate (IPP) and dimethylallyl pyrophosphate (DMAPP), which are important terpenoid compounds that form substrates for the intermediate metabolism of many species and are closely related to gibberellin, abscisic acid, chlorophyll, and quinone synthesis. The IPP pathway is an upstream approach to carotenoids and chlorophyll synthesis, and accelerates the rate of their synthesis, which is consistent with our pigment content result. Mevalonate, geranyl-P, (E, F)-fanesyl-PP, and geranyl geranyl-PP all exhibited upward trends, accelerating the biosynthesis of monoterpene, sesquiterpene, diterpene, and polyterpene, whose expression levels play important roles in biofilm integrity, optical protection, plant growth and development, and electron transfer on the cell membrane.

**Figure 7:**
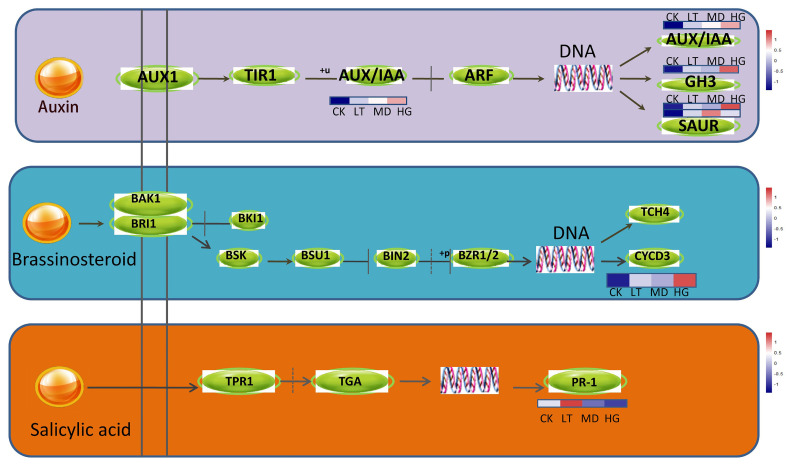
The pathway of plant hormone signal transduction.

Similarly, in the plant hormone signal transduction pathway, auxin, abscisic acid, brassinolide, and salicylic acid were upregulated under shade stress (Figure 7), in genes related to auxin-responsive protein IAA, auxin responsive GH3 gene family, SAUR family protein, and protein phosphatase 2C [EC:3.1.3.16]. These hormones may accelerate the division and proliferation of leaf cells, explaining the phenomenon of increasing leaf area under shade stress, which improves the light energy utilisation ratio.

**Figure 8:**
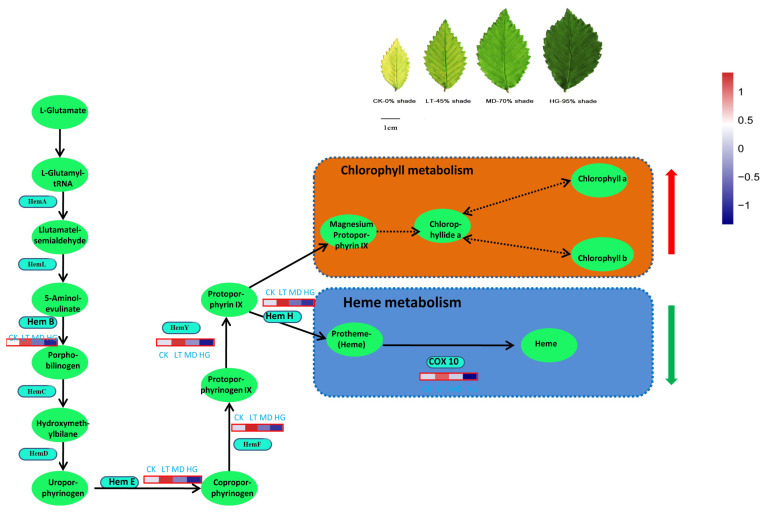
The pathway of Porphyrin and chlorophyll metabolism.

The structures of haeme and chlorophyll were similar, as both were tetrapyrrole compounds, and they both used protoporphyrin IX (proto IX) as a substrate. Among biosynthesis processes, synthesis from ALA to proto IX is common. Proto IX synthesises chlorophyll through the magnesium pathway and generates haeme through the iron pathway. The branching point of tetrapyrrole biosynthesis is an important regulatory site of chlorophyll synthesis, and the direction of proto IX flow directly determines the rate of chlorophyll synthesis. In this study, expression of the key enzyme of haeme synthesis (hemH) showed a downward trend under shade stress, indicating greater proto IX flow to the chlorophyll pathway, which promoted Chla and Chlb synthesis (Figure 8). This process may be the main reason for the observed increase in chlorophyll content.

**Figure 9:**
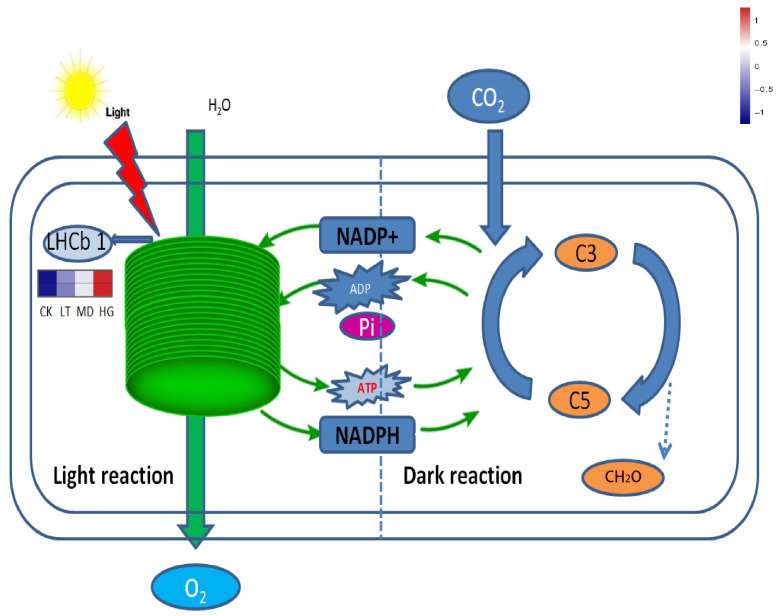
The pathway of photosynthesis.

In the photosynthesis–antenna protein pathway, the expression of light-harvesting complex II Chla/b binding protein (Lhcb1)-related genes was more sensitive to shade stress. *Lhcb1* gene expression increased as shade stress increased, indicating a direct connection between Lhcb1 expression and light illumination changes (Figure 9). Increased Lhcb1 gene expression increases the absorption of light energy and improves light reaction efficiency during photosynthesis.

### 3.7. qPCR Assays

**Figure 10:**
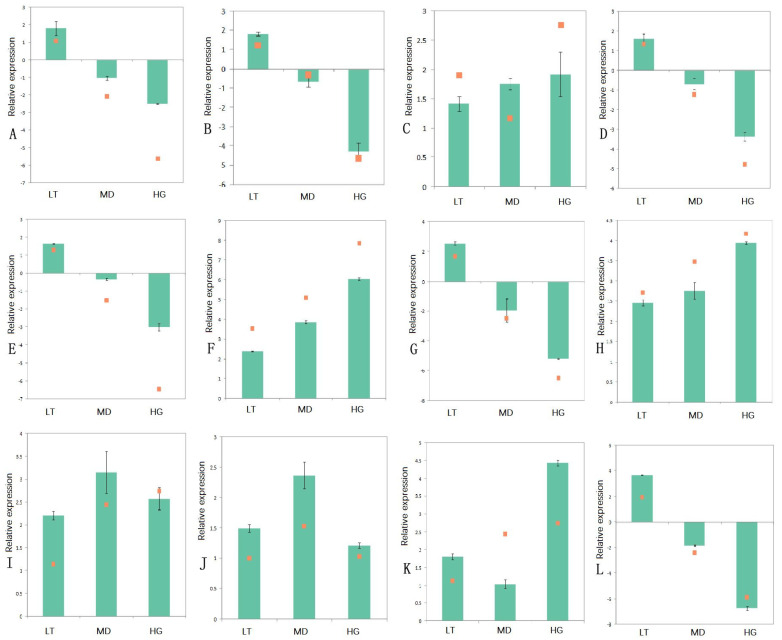
Real-time Q-PCR analysis of 12 differentially expressed genes under different shading treatments. Note: The ordinate is the relative expression (log_2_[T/CK]). (**A**–**L**) denote the relative expression of unigenes 26170, 27871, 27986, 30523, 32570, 7215, 19841, 7389, 39181, 7436, 39536, and 33702, respectively. Real-time Q-PCR was performed for three independent biological replicates, each containing three technical replicates.

To confirm the expression profiles of DEGs identified through RNA sequencing analysis, we randomly selected 12 candidate DEGs for real-time qPCR assays. The quantitative fluorescence results for all genes were consistent with the RNA sequencing results, confirming their accuracy and reliability (Figure 10).

## 4. Discussion and Conclusions

The inclusion of trees with colourful leaves in ornamental gardening has greatly enriched cultural life in some regions. A range of mutants producing colourful leaves has led to the development of a large number of highly valued ornamental varieties. Although mutation mechanisms differ among these varieties, fading and regreening of colourful leaves is a widespread phenomenon that reduces their ornamental value. Numerous studies have shown that leaf colour changes are sensitive to light intensity, such that colours fade at lower light intensity in plants such as *Acer negundo* ‘Aurea’, *U. pumila* ‘Zhonghua Jinye’, and *Berberis thunbergii* var. *Atropurpurea* [6,7,8,9]. Therefore, an understanding of the regulation mechanism of leaf regreening in *U. pumila* ‘Zhonghua Jinye’ under shade stress is particularly important.

In this study, we examined the transcriptome of *U. pumila* ‘Zhonghua Jinye’ under different shade treatments. Our comprehensive analysis showed that under shade stress, *U. pumila* ‘Zhonghua Jinye’ leaf colour loss mainly involved terpenoid backbone biosynthesis and thylakoid grana stacking. Shade stress significantly increased the expression of genes such as terpenoids and linoleic acid, and increased hormone signal transduction. Terpenoids played important roles in plant growth, development, and metabolism. Compounds such as gibberellin, abscisic acid, carotenoids, chlorophyll, steroids, and quinones play important roles in ensuring biofilm system integrity, plant growth and development, and electron transfer on the membrane. In this study, the MVA and DOXP/MEP synthetic pathways showed increasing trends. Genes related to the key rate-limiting enzymes HMGR, MVK, and DXS were upregulated under shade stress, which led to increased IPP and DMAPP contents. The IPP pathway was an upstream approach to carotenoid and chlorophyll synthesis; its accumulation provided a substrate for and accelerates the rate of carotenoid and chlorophyll synthesis, which explained the pigment content increase observed in this study. The expression of DXS, the key enzyme in terpene metabolism, accelerated chlorophyll and carotenoid synthesis and accumulation [21].

The other main reason for leaf regreening in *U. pumila* ‘Zhonghua Jinye’ was chloroplast structural changes. Thylakoid stacking in the grana lamellae changed significantly from a loose state to a closely packed structure under shade stress. In higher plants, almost all chlorophyll and carotenoids combine with proteins to form complexes. The LhcII protein is the major protein on the thylakoid membrane; it combines with approximately 50% of Chla and all of Chlb [22,23,24]. Intracellular chlorophyll and carotenoid are mainly located in the thylakoid membrane, and variation in thylakoid membrane structure directly affects pigment capture and content. Transport, folding and assembly of the LhcII protein also require chlorophyll involvement; thus, the Lhcb1 protein has a close, collaborative relationship with pigment molecules. LhcII has also been proposed to play a key role in cation-mediated formation of thylakoidal grana stacks [25]. Lhcb1 protein increases can maintain the stability of the chloroplast thylakoid structure, which promotes close stacking in the grana lamellae and, combined with more pigment molecules, eventually leads to increased pigment accumulation and photosynthetic efficiency. Labate [26] transferred the pea *Lhcb1* gene into tobacco and found that its expression increased the number of thylakoid membranes per chloroplast, grana stacking density, chloroplast numbers per palisade cell and chlorophyll content, which were consistent with our results. Pietrzykowska [27] examined the RNA interference expression vector of the *Lhcb1* gene in *Arabidopsis thaliana* and found that it developed yellow leaves, with decreased grana lamella stacking layers; these results were also consistent with our findings. Plant pigments are located mainly in thylakoids; therefore, the stacking state within the grana lamellae is closely related to pigment accumulation. In this study, the change from loose thylakoid stacking to closely packed structures under shade conditions eventually led to increased pigment accumulation and photosynthetic efficiency.

This study is the first to report a self-regulation mechanism underlying *U. pumila* ‘Zhonghua Jinye’ leaf colour changes under shade stress. However, the signal transduction process related to this mechanism remains unclear. Whether plant hormones are involved in leaf regreening in this variety is also unknown. Therefore, signal transduction and Lhcb1 protein synthesis and transmembrane localisation must be further studied. The results of this study provide a theoretical basis for pigment metabolism in leaf colour mutant plants.

## Figures and Tables

**Table 1:** Unigene annotation statistical table.

Annotation Database	Annotated Number	300 ≤ Length < 1000	Length ≥ 1000	Frequency
Nr	30,802	9553	15,520	48.93%
Pfam	24,272	6831	14,338	38.56%
KOG	20,861	6240	11,171	33.14%
Swissprot	19,220	5639	11,063	30.53%
GO	17,535	5134	8882	27.86%
KEGG	12,983	3880	7011	20.63%
COG	11,990	3038	6950	19.05%
All	33,023	10,543	16,149	52.46%

**Table 2:** DEG unigene numbers in different pathways of shade stress.

Pathways	Related Unigene	DEG Unigene			Enrichment Factor			*p*-Value		
		LT	MD	HG	LT	MD	HG	LT	MD	HG
Calcium signalling pathway	94	2	10	29	3.27	2.36	0.00	0.00	0.00	3.27
Carbon metabolism	643	2	18	66	5.89	0.78	0.00	0.78	1.00	5.89
cGMP-PKG signalling pathway	149	2	10	33	3.27	1.69	0.02	0.02	0.01	3.27
Cutin, suberine and wax biosynthesis	37	3	2	5	0.65	1.03	0.02	0.34	0.65	0.65
Cyanoamino acid metabolism	67	2	3	7	0.98	0.80	0.02	0.37	0.88	0.98
Glutathione metabolism	133	2	4	16	1.31	0.92	0.05	0.63	0.84	1.31
Glycerolipid metabolism	116	2	7	19	2.29	1.25	0.05	0.08	0.33	2.29
Glycolysis/Gluconeogenesis	315	3	12	27	3.92	0.65	0.06	0.33	1.00	3.92
Inositol phosphate metabolism	103	2	3	18	0.98	1.33	0.08	0.66	0.24	0.98
Methane metabolism	178	2	6	15	1.96	0.64	0.10	0.52	1.00	1.96
Peroxisome	181	4	5	26	1.64	1.10	0.12	0.71	0.57	1.64
Phenylpropanoid biosynthesis	171	2	5	11	1.64	0.49	0.13	0.66	1.00	1.64
Pyruvate metabolism	254	2	11	23	3.60	0.69	0.22	0.21	1.00	3.60
Starch and sucrose metabolism	265	2	7	21	2.29	0.61	0.24	0.77	1.00	2.29
Tryptophan metabolism	99	2	8	16	2.62	1.23	0.67	0.02	0.37	2.62

## Data Availability

The original contributions presented in this study are included in the article/Supplementary Material. Further inquiries can be directed to the corresponding authors.

## Supplementary Materials

The following supporting information can be downloaded at https://www.mdpi.com/article/10.3390/plants14182868/s1: Figure S1: The GO function enrichment of profile 25 and profile 22; Figure S2: The KEGG enrichment; Table S1: Primer information.
