# Supplementary material for: Analysis of the Metabolic and Structural Changes in Ulmus pumila ‘Zhonghua Jinye’ Leaf Under Shade Stress
Source: Plants (Basel). 2025 Sep 15;14(18):2868. doi: 10.3390/plants14182868 (PMC12473242; doi:10.3390/plants14182868)

Table S1. Primer information

Primer	Sequence
c26170F	F:CAATCTGTCTGGATAAAGTAGGCAATG
c26170R	R:CAGGTGCAACCATAATCATCAGC
c27871F	F:TTTTGATAGAGCGGACAAACCTC
c27871R	R:GTACGTCTACCTGTCGGTATTGC
c27986F	F:AGAAAGGAGGCTTCAAAGGGATA
c27986R	R:GAATCTTCACCGTCCGTTGTTAT
c30523F	F:GCTACTGAAGATGGCAAGACTAA
c30523R	R:GTCGCACAACGAATGGAACTCTG
c32570F	F:TCACTTCTAAATTCCGTCCTCAG
c32570R	R:CACGGTCGATACTATCCACTCTT
c7215F	F:TGACCACTTGGCTGACCCTGTTA
c7215R	R:TCCCAACCCTCTAGGAGGTGCTT
c19841F	F:TTGATGCCGACGGTTCTGGTGAG
c19841R	R:CTTGCATGGCCTCGGTATCTTCC
c7389F	F:TGATGAACATGGCATTGATACAAG
c7389R	R:GTTCGAGGTCCATAAGAACAGCT
c39181F	F:CTAAAGGGCATTACACCGAAGGA
c39181R	R:GAGAATGGCAAATCTGGAAACCT
c7436F	F:CTCGCATCATTGTAGCAGTAGCC
c7436R	R:ACTGGGAGTTTCTTAGGCGTTGT
c69536F	F:GCTGCCTGGAACTACGCCAAACT
c69536R	R:TCTGTAATCGCTACTGCGACAAT
c33702F	F:TCCCTTCTTCATCCTCGTACCCA
c33702R	R:CACAACGACTACCACCACGACAA

Figure S1. The GO function enrichment of profile 25 and profile 22.

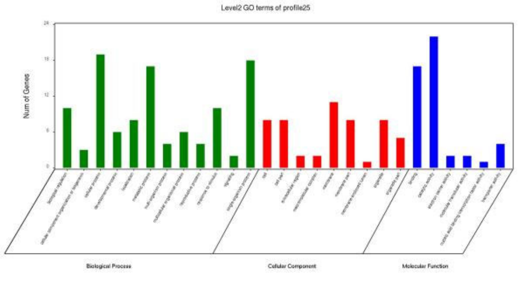

Profile 25

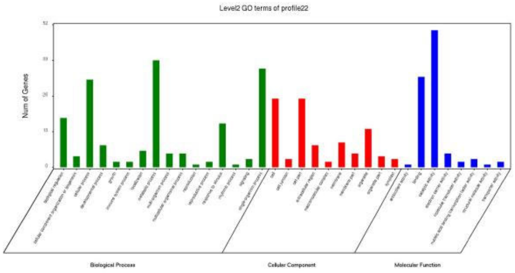

Profile 22

Figure S2. The KEGG enrichment.

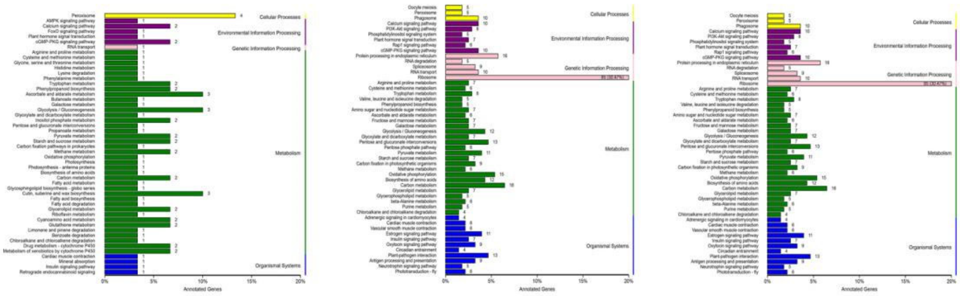

Supplement: Supplementary file 1 [file plants-14-02868-s001.zip › plants-3782476-supplementary.pdf]
